# Supplementary material for: Whole-Genome Resequencing of Experimental Populations Reveals Polygenic Basis of Egg-Size Variation in Drosophila melanogaster
Source: Mol Biol Evol. 2015 Jun 3;32(10):2616–32. doi: 10.1093/molbev/msv136 (PMC4576704; doi:10.1093/molbev/msv136)
Supplement: Supplementary Data [file supp_32_10_2616__index.html]

Whole-Genome Resequencing of Experimental Populations Reveals Polygenic Basis of Egg-Size Variation in Drosophila melanogaster — Whole-Genome Resequencing of Experimental Populations Reveals Polygenic Basis of Egg-Size Variation in Drosophila melanogaster — Supplementary Data 

# Whole-Genome Resequencing of Experimental Populations Reveals Polygenic Basis of Egg-Size Variation in *Drosophila melanogaster*

## Supplementary Data

files

- Supplementary Data - xlsx file
- Supplementary Data - pdf file
