# Supplementary material for: Whole-Genome Resequencing of Experimental Populations Reveals Polygenic Basis of Egg-Size Variation in Drosophila melanogaster
Source: Mol Biol Evol. 2015 Jun 3;32(10):2616–32. doi: 10.1093/molbev/msv136 (PMC4576704; doi:10.1093/molbev/msv136)
Supplement: Supplementary Data [file supp_msv136_Supplementary_Figures.pdf]

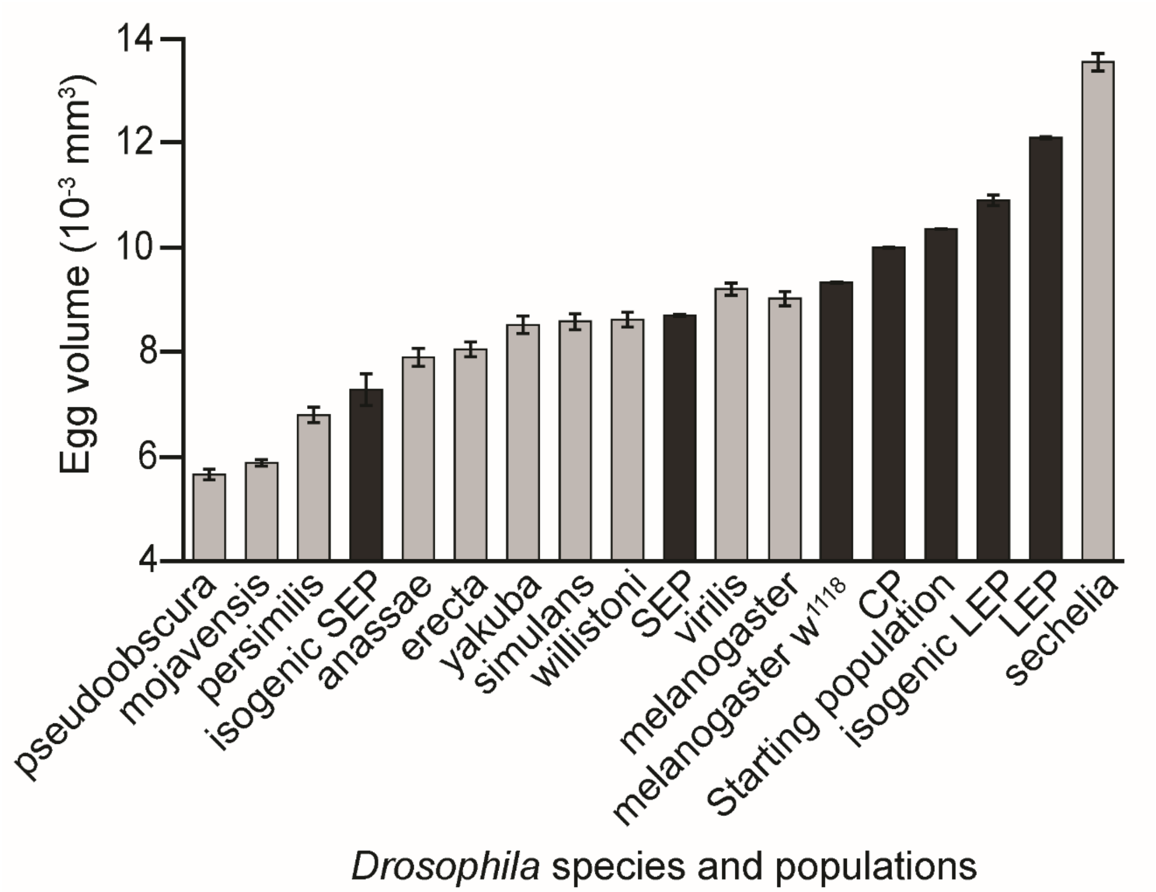

Supplementary Figure 1. Egg sizes in *Drosophila* species and populations. Comparison of egg volumes of the evolved populations to those from diverse *Drosophila* species. Grey bars indicate egg size for various species that were obtained from a previous study (Markow, et al. 2009). Eggs sizes for various populations of *Drosophila melanogaster* involved in this study are shown in black bars. Eggs of females from the control populations at the end of the selection experiment were similar to those from the starting populations. Isogenic lines derived from LEP and SEP cages retained the changes in egg sizes in the respective directions after 40 generations of inbreeding. Egg volumes for isogenic lines were calculated as the average of the five lines in Figure 2D.Error bars indicate SEM.

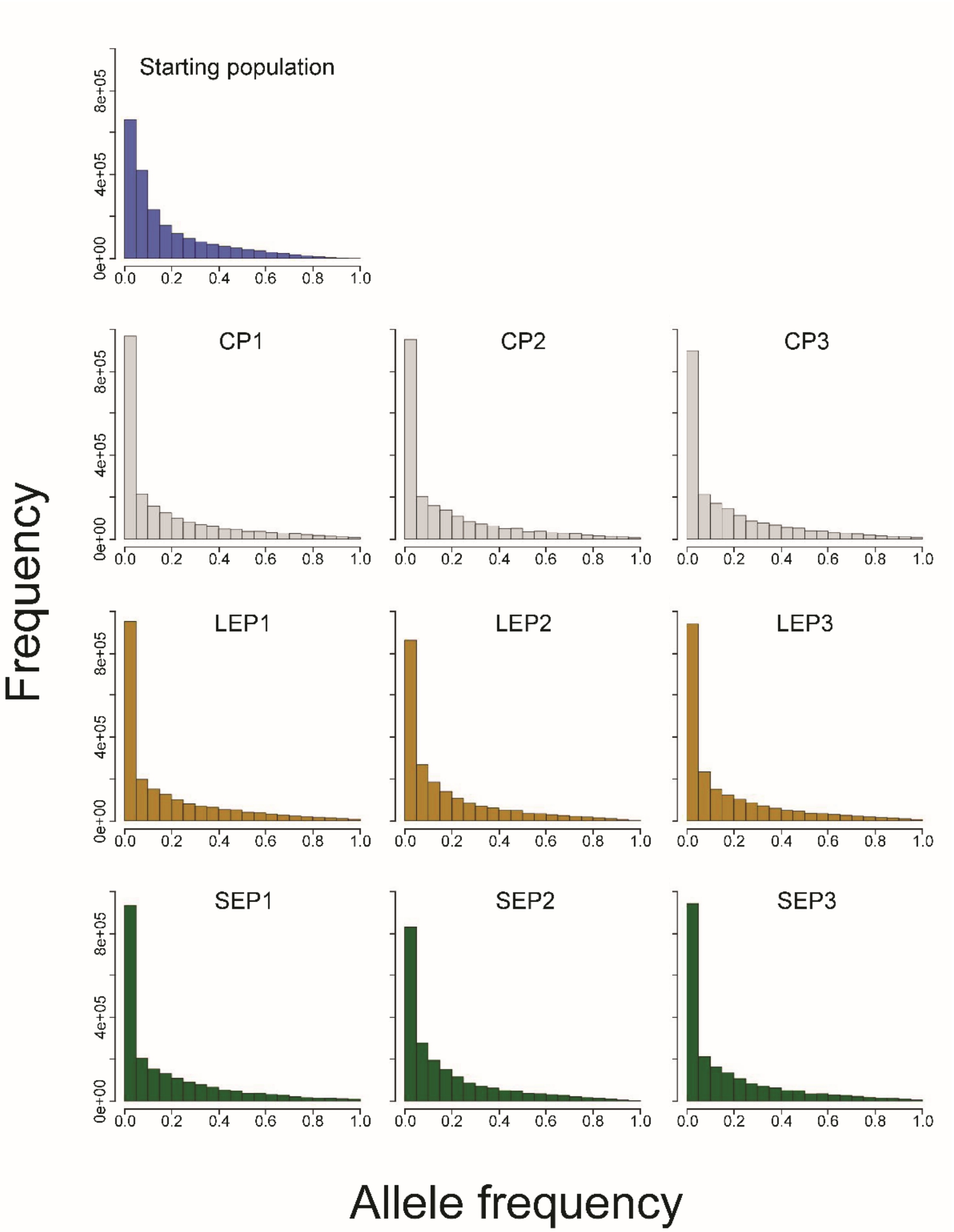

Supplementary Figure 2. Allele frequency spectrum of variant alleles in all ten populations sequenced. Allele frequency of variant alleles in the starting population (blue) and three replicates of controls (grey), large (yellow), and small (green) are shown.

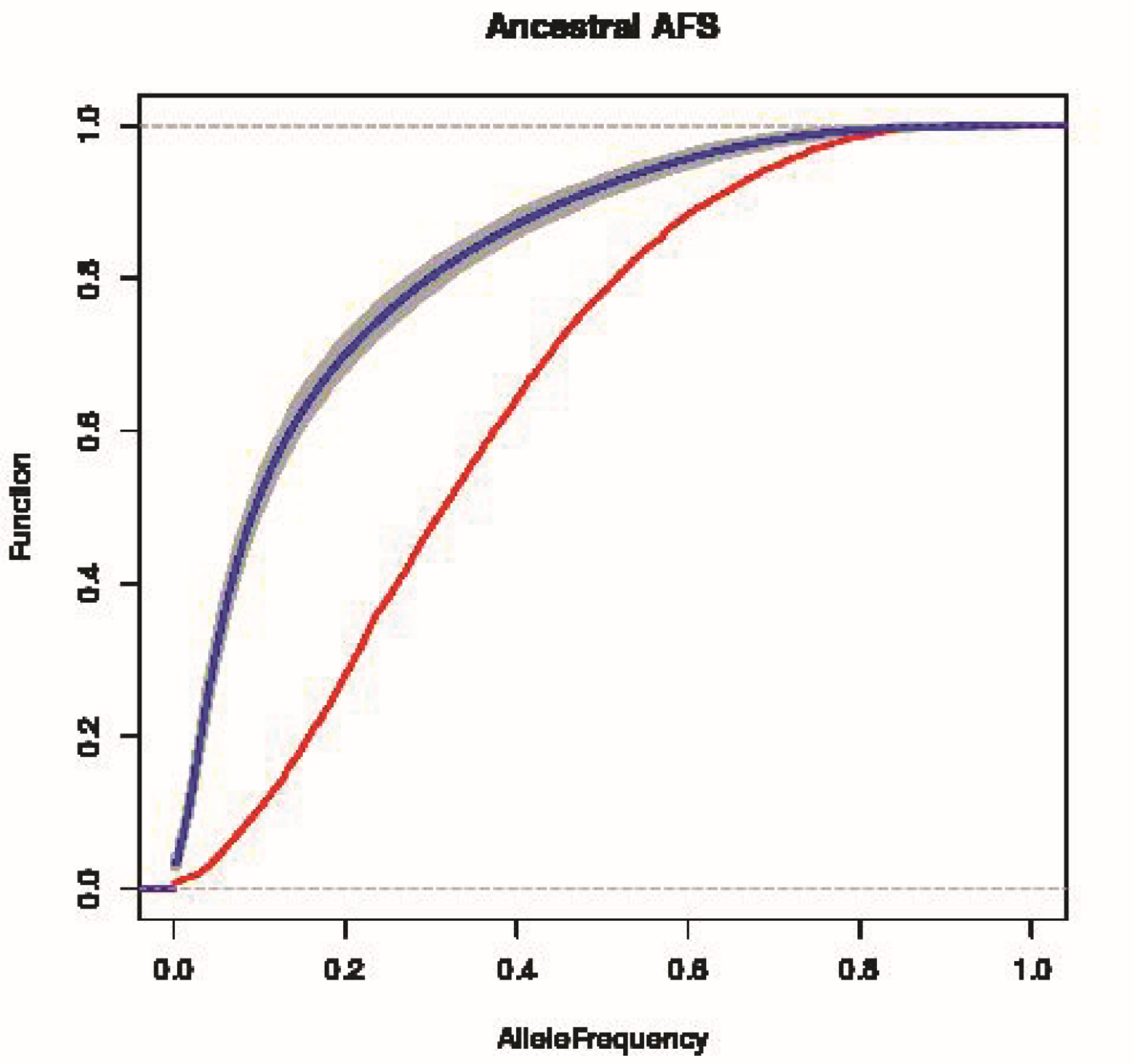

Supplementary Figure 3. Cumulative allele frequency spectrum of SDV in ancestral population. Cumulative frequency spectrum of significantly diverged variants (in red), all non-significant variants (in blue) and 1000 sets of 4,137 randomly sampled non-significant variants (in grey) in starting population.

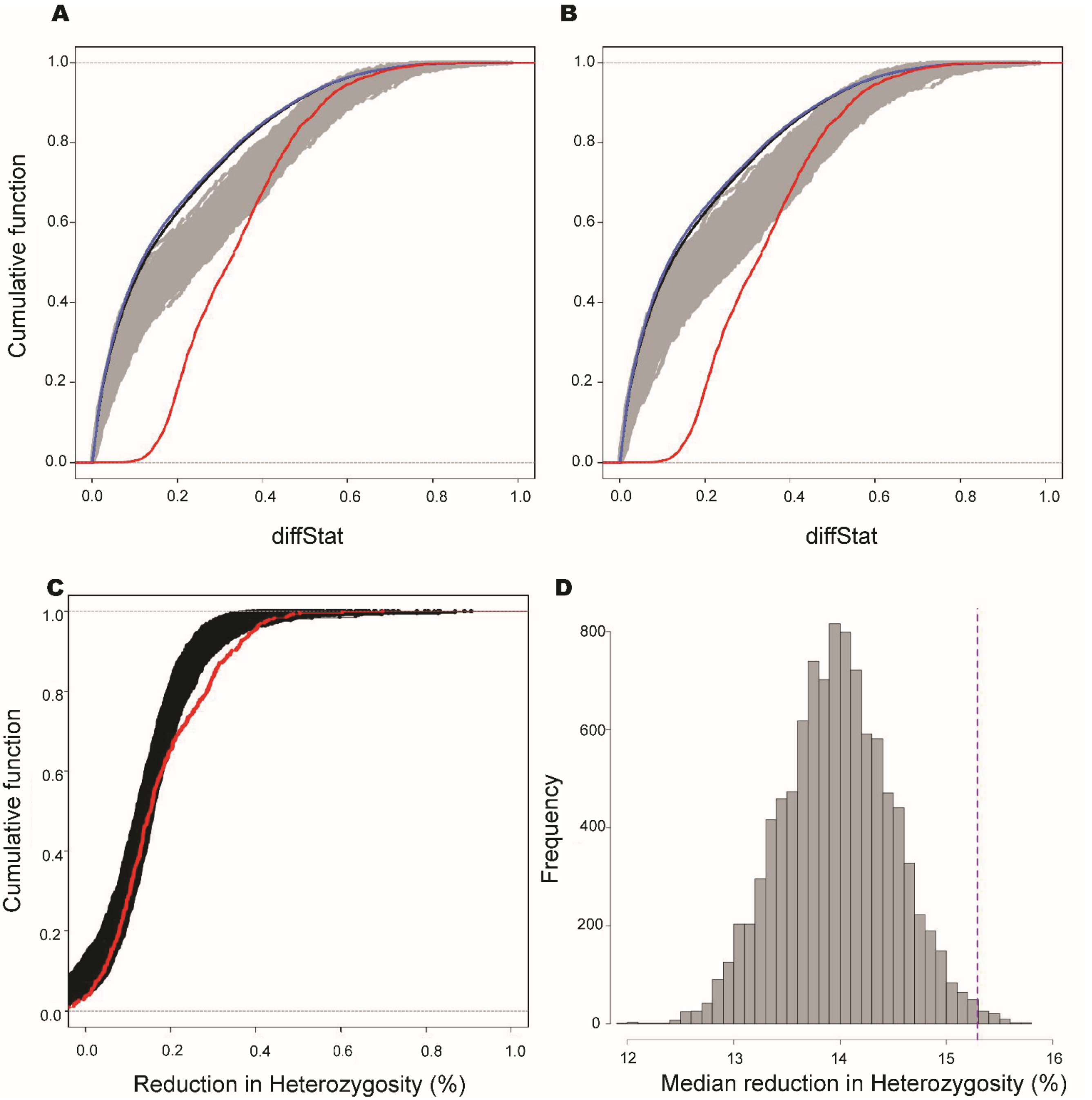

Supplementary Figure 4. Permutation test for diffStat statistic and heterozygosity. A) Comparison of diffStat statistic of SDV (in red) and randomly selected variants matched by chromosomal location (in grey). Genomewide diffStat scores and that of non-SDV are also shown in black and blue respectively. **B)** Comparison of diffStat statistic of SDV (in red) and randomly selected variants matched by chromosomal location and starting allele frequency (in grey). In each case, ten thousand sets of 4,137 variants were randomly selected with replacement. **C)** Comparison of reduction in heterozygosity relative to the starting population in 415 significant windows in LEP (red) with that in ten thousand sets of equal number of randomly sampled windows matched by chromosomal arm in CP (black). **D)** Distribution of median reduction in heterozygosity in each set of randomly sampled windows relative to the starting population. Dotted purple line indicates the median reduction in heterozygosity in the 415 significant windows in the LEP. Only 66 out of 10,000 permuted sets showed stronger reduction in median heterozygosity (P = 66/10000 = $6.6e^{-03}$).

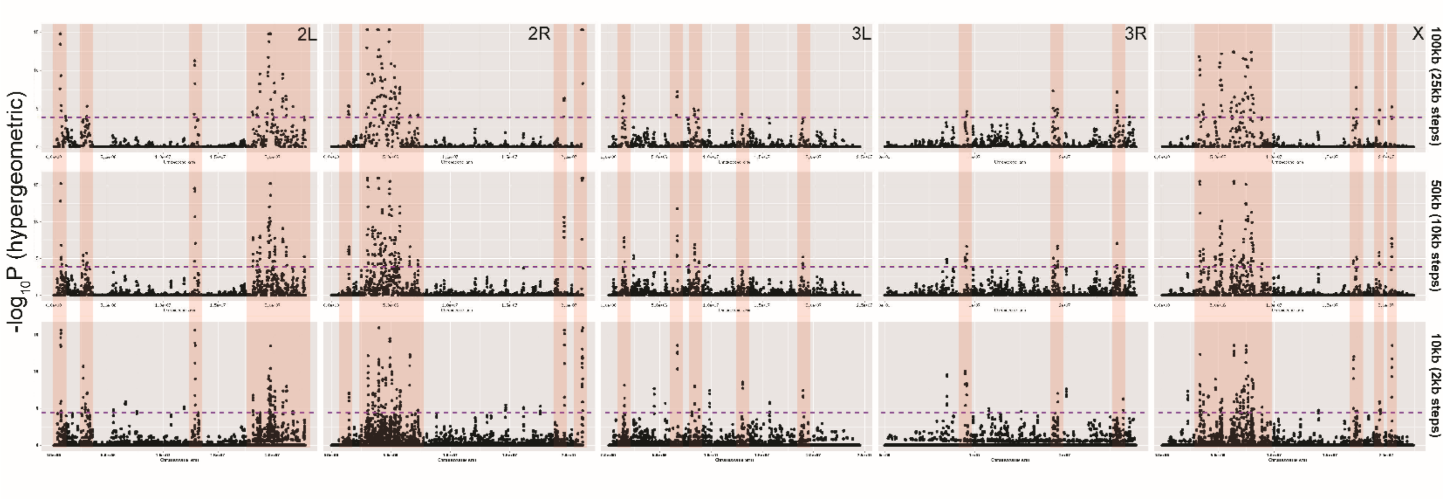

Supplementary Figure 5. Distribution of SDB for various window sizes in the *Drosophila* genome. Top row: SDB in 100kb windows with 25kb steps, middle row: SDB in 50kb windows with 10kb steps, bottom row: SDB in 10kb windows with 2kb steps. Dotted purple lines indicate genomewide significance threshold for multiple testing. Regions highlighted in orange show peaks that are significant for all window sizes.

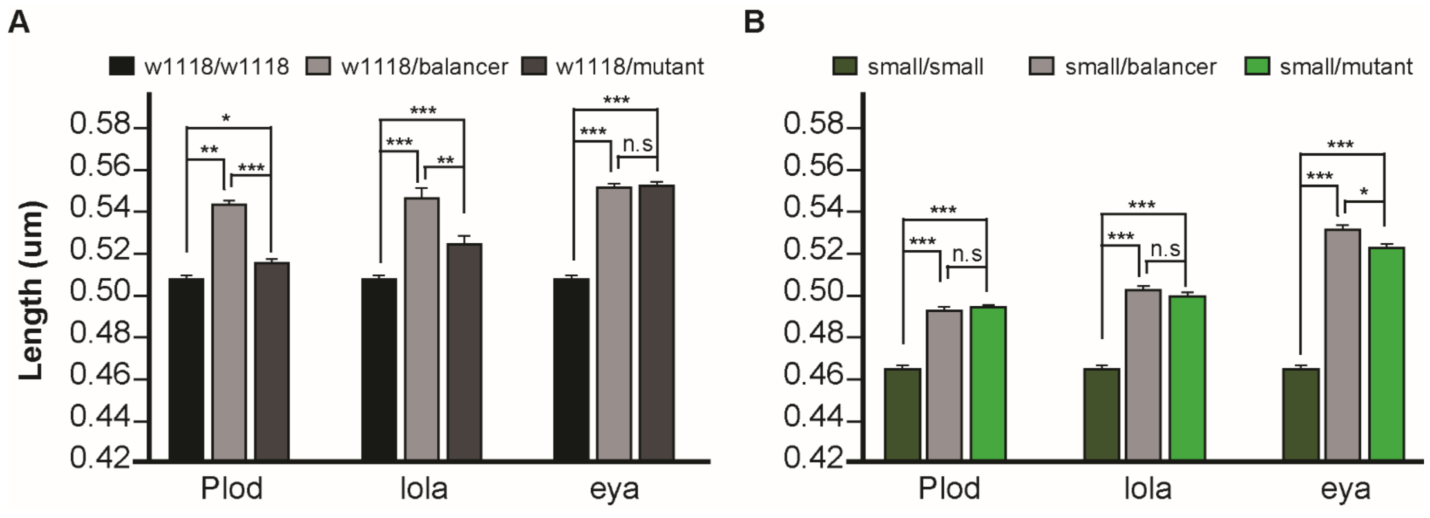

Supplementary Figure 6. Genetic crosses show complexity of egg size in flies. Three genes were tested for their effects on egg length in **(A)** a common laboratory w^{1118} lines and **(B)** an isogenic line derived from small populations. Comparison of egg length from isogenic females in each of the genetic background (black and dark green for w^{1118} and small, respectively) to those carrying one copy of the balancer chromosome (light grey) indicated that egg length can vary simply by changing the genetic background via introduction of balancer chromosomes. Females carrying chromosomes with mutant alleles of each of the candidate genes (dark grey and light green for w^{1118} and small, respectively) also increased egg lengths, however the effect was not larger than that of the balancer chromosome in these genetic backgrounds. *P<0.005, **P<0.001, ***P<0.0001.
